# HIV-1 and cocaine disrupt dopamine reuptake and medium spiny neurons in female rat striatum

**DOI:** 10.1371/journal.pone.0188404

**Published:** 2017-11-27

**Authors:** Mehrak Javadi-Paydar, Robert F. Roscoe, Adam R. Denton, Charles F. Mactutus, Rosemarie M. Booze

**Affiliations:** Behavioral Neuroscience Laboratory, Department of Psychology, University of South Carolina, Columbia, South Carolina, United States of America; University of Missouri Kansas City, UNITED STATES

## Abstract

HIV-1 and addictive drugs, such as cocaine (COC), may act in combination to produce serious neurological complications. In the present experiments, striatal brain slices from HIV-1 transgenic (Tg) and F344 control female rats were studied. First, we examined dopamine (DA) reuptake in control, HIV-1, COC-treated (5µM) and HIV-1+COC-treated, striatal slices using fast scan cyclic voltammetry. COC-treated striatal slices from F344 control animals significantly increased DA reuptake time (T80), relative to untreated control slices. In contrast, in HIV-1 Tg striatal slices, DA reuptake time was extended by HIV-1, which was not further altered by COC treatment. Second, analysis of medium spiny neuronal populations from striatal brain slices found that controls treated with cocaine displayed increases in spine length, whereas cocaine treated HIV-1 slices displayed decreased spine length. Taken together, the current study provides evidence for dysfunction of the dopamine transporter (DAT) in mediating DA reuptake in HIV-1 Tg rats and limited responses to acute COC exposure. Collectively, dysfunction of the DAT reuptake and altered dendritic spine morphology of the MSNs, suggest a functional disruption of the dopamine system within the HIV-1 Tg rat.

## Introduction

HIV associated neurocognitive disorders (HAND) affect approximately half of the HIV seropositive population [1,2,3], with affected women demonstrating increased neurocognitive impairments relative to affected men [4].Furthermore, co-morbid drug abuse is often observed in women living with HAND [1,2].With these observations in mind, the present study examined the effects of cocaine treatment upon HIV-1 Tg female rats in order to potentially determine neuronal substrates of HAND in female drug abusers. Our lab has previously reported that HIV-1 Tg female rats have compromised dendritic spine morphology relative to HIV-1Tg males and F344 controls [3]. Specifically, medium spiny neurons (MSN) in the nucleus accumbens region of transgenic females were found to have an increased frequency of shorter, stubbier dendritic spines, although the reasons for these dendritic spine alterations are remain unclear.

Direct infusion of the HIV-1 protein Tat into the nucleus accumbens in animals with prior chronic cocaine experience produces both a hyperdopaminergic basal tone [5]as well as behavioral alterations [6]. Interestingly, acute treatment with HIV-1 Tat protein alone does not significantly alter DA levels. However, prior *in vivo* cocaine exposure produces noticeable dopaminergic alterations. [5] Our lab has previously reported that the HIV-1 Tat protein binds to and directly inhibits the dopamine transporter (DAT) in vitro [7–10]. Tat produces conformational changes in the DAT protein, which may alter the affinity of cocaine for the DAT [7,8]. Similarly, the HIV-1 gp120 protein also impairs the function of the DAT in dopaminergic neurons [11]. Collectively, HIV-1 proteins (such as Tat and/or gp120) combined with cocaine exposure produce increases in extracellular DA concentrations.

Such increases in extracellular dopamine over a prolonged period may produce an inflammatory neuronal environment, thereby altering neuronal microstructures such as dendritic spines and may lead to eventual loss of dopaminergic nerve terminals [12]. Vera et al. (2016) has recently demonstrated increased neuroinflammation in subcortical gray matter in the basal ganglia of adult HIV-1 subjects [13]. However, reports of neuroinflammation in the HIV-1 Tg rat have been inconsistent with both increases and no changes reported [14–16]. Our lab has found no changes in chemokines/cytokine expression in adulthood following neonatal HIV-1Tat protein injection [17].

Given that neuroinflammation has detrimental effects on dendritic spine morphology in degenerative diseases [18–20], including viral infection [21], mechanisms of synaptic alterations in the HIV-1 Tg rat [3] are of interest. The interactive effects of HIV-1 Tat protein and cocaine upon synaptic integrity *in vitro* [22] also suggest that HIV-1 proteins + cocaine may alter dendritic spines. Shifts in dendritic spines, as a result of cocaine and HIV-1 interactions, may be one key neuropathological change in the striatum/nucleus accumbens region during HIV-1 infection.

In the present experiments, we used striatal brain slices from female HIV-1 Tg and F344 control rats to assess the function of DAT (both with and without cocaine) and subsequent structural alterations in dendritic spines. Evidence obtained using fast scan cyclic voltammetry (FSCV) strongly supports the hypothesis of alterations of the DA reuptake system within the striatum in female HIV-1 transgenic rats. The synaptodendritic pathology in the HIV-1 transgenic rats striatal slices exposed to cocaine show alterations as previously reported for isolated neurons [22] and for intact HIV-1 transgenic rats [3]. These findings provide insight into pathophysiological alterations present in HIV-1 and comorbid drug abuse.

## Materials and methods

### Ethics statement

Experiments were conducted in accordance with the recommendations in the Guide for the Care and Use of Laboratory Animals of the National Institutes of Health. The research protocols were approved by the Institutional Animal Care and Use Committee at the University of South Carolina (assurance number: A3049-01).

### Animals and preparation of striatal brain slices

HIV-1 Tg females (n = 10) and control female Fisher 344 rats (n = 8) (130–160 g at the time of sacrifice) were obtained from Envigo, Inc. (Indianapolis, IN). The animal facility was maintained at targeted conditions of 21°±2°C, 50%±10% relative humidity and had a 12-h light:12-h dark cycle, with lights on at 0700 h (EST). Animals had access to food and water *ad libitum* and were housed two per cage throughout the experiment. Vaginal lavage was performed daily to evaluate both estrous cycle length and estrous stage. Smears obtained were immediately evaluated under a 10 X light microscope and cell morphology was correlated to cycle state [3,23]. Two complete, consecutive, estrous cycles were required prior to sacrifice.

Female rats in diestrus were euthanized (Sevoflurane overdose) and their brains were rapidly removed and chilled in ice-cold aCSF (124 mM NaCl, 5 mM KCl, 1.5 mM MgCl_2_, 2.5 mM CaCl_2_, 1.4 mM NaH_2_PO_4_ anhydrous, 10 mM dextrose and 26 mM NaHCO_3_), which was continuously bubbled with a 95% O_2_/5% CO_2_ mixture, pH adjusted to 7.4. Brains were mounted in a chilled, aCSF-filled chamber, and coronal brain slices (400 μm) were obtained using a vibratome (Series 1000; Technical Products International, St. Louis, MO). Striatal slices (AP + 1.0 mm, ML +/-2.3 mm, DV -4.0 to -6.0 mm; 0.5 mm increments) were incubated in oxygenated aCSF at 22°C for at least one hour prior to electrochemical recordings. During recordings, slices were continuously superfused with oxygenated aCSF solution at a rate of 2 ml/min. All recordings were carried out at a bath temperature of 32°-33°C.

### Electrochemical recordings in striatal brain slices

In vitro FAST-scan cyclic voltammetry was performed using Nafion-coated carbon-fiber microelectrodes (length 150–200 μm, The Center for Microelectrode Technology, University of Kentucky College of Medicine, KY). Single carbon-fiber type electrochemical working microelectrodes (30 μm O.D., length 150–200 μm) were used to measure DA release signals. Prior to use, all microelectrodes were calibrated *in vitro* with a known concentration of DA to determine their selectivity, sensitivity, and reduction/oxidation current responses to DA. Microelectrodes had an average selectivity of >900:1 for DA over 3, 4-dihydroxyphenylacetic acid (DOPAC) or ascorbic acid. Dopamine calibrations were averaged to convert a signal (in nanoamps) of DA to micromolar concentration of DA. Each microelectrode displayed linear responses to DA within the concentrations used [24,25]. Reduction/oxidation current ratios with an average range of0.5 to 0.7 were produced, indicating that the microelectrode was detecting DA and not ascorbic acid [24]. The limit of detection for DA was typically 25 nM. DA was applied directly to the striatal slices using a picospritzer at concentrations ranging from 0.2–3.0 μM.

Microelectrodes were connected to a headstage (Quanteon, L.L.C. Nicholasville, KY) with a gold-plated Amphenol wire-crimp (Mill-Max®, Oyster Bay, New York). The FAST-12 system (Fast Analytic Sensing Technology, Quanteon, L.L.C. Nicholasville, KY) was used to perform chronoamperometric measurements (5 Hz) as previously described for the detection of dopamine [24,26]. Baseline measurements were taken following a 1 hour stabilization period. Cocaine (5 μM) was superfused at a constant rate of 2 ml/min for at least 10 min before electrochemical recordings [27,28].

DA reuptake rates and dose response of signals in the presence/absence of cocaine were assessed to determine the DAT activity in HIV-1 transgenic rats. For each individual signal, the following parameters were analyzed: (1) peak amplitude of the obtained signal; (2) T80, seconds between peak maximum and time where level has decreased 80% from maximum; and (3) clearance rate (Tc, in mM/sec), defined by the change in DA amplitude between the T20 and T60 time points (e.g., the slope of the linear portion of the decay curve). These parameters were chosen as they are known to primarily reflect DA uptake, rather than metabolism or diffusion [25].

### Diolistic labeling of medium spiny neurons

Immediately following the recording session, diolistic labeling was performed on the striatal tissue slices (n = 4–5 animals) [21,29], as previously described [3]. Approximately 300mg of tungsten beads were dissolved in 99.5% pure methylene chloride prior to use. Crystallized DiI was dissolved in methylene chloride, vortexed, and protected from light. Following sonication, 100 μl of the bead solution was placed on a glass slide and 150 μl of the DiI solution was titrated on top, briefly mixed with a pipette tip, and allowed to air dry directly on the slide. After air-drying, a razor blade and wax paper was used to collect the dye/bead mixture into a 15 ml conical tube (BD Falcon, San Jose, California). Next, 3 ml ddH2O was added to the tube which was subsequently sonicated (Branson Sonifier 150, Branson Ultrasonics, Danbury CT) for 10 minutes.

Tefzel tubing (IDEX Health Sciences, Oak Harbor, WA) was cut into 1.7 M segments in preparation for bullet coating. Polyvinylpyrrolidone was dissolved in 10 ml ddH2O, strongly vortexed, then passed through each length of tubing. The 3 ml of bead/dye solution was slowly drawn into the tubing and placed in the spinning tubing prep station (Bio-Rad) for 5 minutes. After removing the water from the tube, the dry tubing was spun in the prep station for approximately 10 minutes with a nitrogen gas flow of 1.0 LPM. Nitrogen gas flow through the tubing was adjusted to 0.4–0.5 LPM and the tubing was further spun for 20–30 minutes to ensure that the tubing was fully dry. Next, tubing was cut into 13 mm segments/cartridges using a supplied tubing cutter (Bio-Rad) and stored under anhydrous conditions until use.

A ballistic delivery device (Gene gun, Bio-Rad, Hercules, CA) was loaded with the 13 mm cartridges. Tissue slices were taken from the aCSF bubbling chamber, immediately fixed in 4% paraformaldehyde for 10 minutes, then placed in PBS for two 5-minute washes. Helium gas flow was adjusted to 80 PSI, and particles were then ballistically expelled through 3 μm pore filter paper (Merck Millipore, ISOPORE filters, Carrigtwohill, CO) directly onto the slice, with the barrel placed approximately 2.5 cm away from the sample. After one thorough 15 minute wash in phosphate buffered saline (PBS), sections were stored overnight at 4˚ C to allow for adequate dye diffusion into the neuronal membrane. Sections were mounted using Pro-Long Gold Antifade, coverslipped (#1 coverslip; ThermoFisher Scientific, Waltham, MA), and stored in the dark at 4˚ C.

### Medium spiny neuron (MSN) spine quantification

MSN spine quantification was performed on coronal brain slices from the nucleus accumbens core region (2.28 mm to 0.60 mm anterior to bregma). Neurons with bright, continuous dendritic staining extending from the soma, minimal diffusion of the DiI into the extracellular space, and low background/dye clusters were selected for spine analysis. Z-stack images were obtained with a Nikon TE-2000E confocal microscope utilizing Nikon’s EZ-C1 software (version 3.81b). Dendritic spine imaging was performed at 60 X (n.a. = 1.4) with Z plane intervals of 0.15–0.25 μm (pinhole size 30 μm; backprojected pinhole radius 167 nm). A green helium-neon (HeNe) laser with a wavelength emission of 543 nm was used for DiI fluorophore excitation. Morphometric analysis of spines was performed using Neurolucida version 11.01, coupled with the AutoNeuron and AutoSpine analysis extension modules (MicroBrightField, Williston, VT).

Dendritic spine parameters used were adapted from Yuste, 2011. [30] Specifically, any spine with a length greater than 5 μm was removed from the analysis, as these were considered to be indicative of thin filopodia [3,30,31]. Neurons with continuous staining and defined dendritic branching were chosen for analysis (n = 4–5 animals/group, 1–2 neurons/animal).

### Drugs and chemicals

Dopamine, cocaine HCl, polyvinylpyrrolidone, and 99.5% methylene chloride were purchased from Sigma-Aldrich (St. Louis, MO). Crystallized DiI and Pro-Long Gold Antifade mounting medium were purchased from Invitrogen (Carlsbad, CA). Tungsten microcarrier beads were purchased from Bio-Rad (Hercules, CA).

### Statistical analysis

Concentration- and time-response curves were analyzed and compared using GraphPad (Prism V5). Dendritic spine parameters, such as length were compared by their population distribution using a chi-squared test. For all tests, P < 0.05 was defined as statistically significant.

## Results

### DA reuptake in HIV-1 Tg and control rat striatum

**Fig 1:**
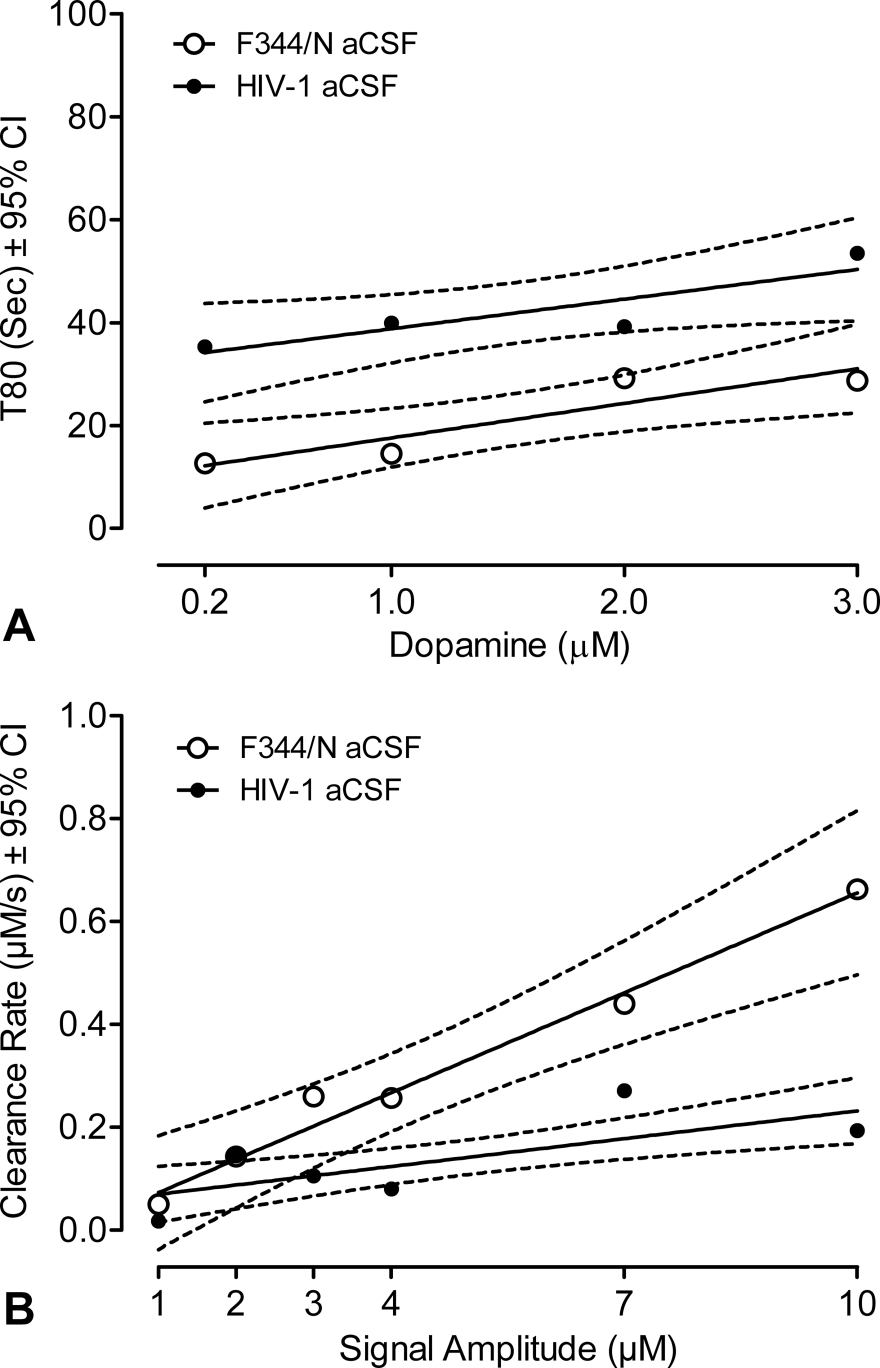
Dopaminergic reuptake is inhibited in HIV-1 Tg striatal slices. **(A)** Mean T80 values are presented as a function dopamine concentration for F334/N control and HIV-1 transgenic groups. The elevations (intercepts) of the curves were significantly (p ≤ 0.002) different between the control and HIV-1, indicating that dose response were slower in the HIV-1 over the tested range of amplitudes. **(B)** Mean clearance rates are presented as a function of signal amplitude for F344/N control and HIV-1 transgenic groups. Clearance rates were linear with respect to amplitude over this amplitude range. However, rates of clearance were significantly lower in the HIV-1 rats (slope difference, p ≤ 0.001). Data are represented for 10 HIV-1 and 8 control animals; all recordings from each animal were averaged together for the analysis of that animal. aCSF stands for artificial cerebral spinal fluid.

As shown in Fig 1A electrochemical signals for DA reuptake were of significantly greater amplitude throughout the dose range investigated (~3X, β_0_ = 10.9±9.0 vs. 33.0±10.5, X±95%CI) in HIV-1 Tg rats relative to controls (F_1, 88_ = 10.5, p≤0.002). The slopes of the dose-response functions for DA reuptake, however, did not differ as a function of the HIV-1 transgene (β_1_ = 6.7±4.8 vs. 5.8±5.6, X±95%CI). DA clearance occurred as a linear function of signal amplitude in the striatum of both control and HIV-1 Tg rats (r^2^ = 0.98 and 0.82 for the control and HIV-1 Tg, respectively) with the slope of the line significantly more shallow in HIV-1 Tg slices relative to controls (4X, β_1_ = 0.018±0.011 vs. 0.065±0.025, X±95%CI; F _1, 80_ = 13.0, p ≤ 0.001), indicating a reduced clearance rate over the entire amplitude range.

### DA reuptake in HIV-1 Tg and control rat striatum exposed to cocaine

The results outlined above suggest that DA clearance differs between control and HIV-1 Tg rats in a manner consistent with known differences in DAT function. However, to more conclusively demonstrate the role of the DAT in mediating the lower functionality of the DA electrochemical signal in HIV-1 Tg rats, we used the uptake inhibitor cocaine, which produces neurobiological effects mostly due to inhibition of DAT function[24,25], in order to test DAT function.

DA was first locally applied to obtain reproducible electrochemical signals. After establishing a stable DA signal, cocaine (5 μM) was superfused into the slice chamber for 10 minutes. DA clearance was then monitored during and after bath application of cocaine. Average base-line signals were 2 μM in amplitude in all experiments, which is consistent with previous findings that DA signals in this concentration range are modulated by uptake inhibitors [26,27]. We then compared the effects of cocaine on DA electrochemical signals in the stratum of both HIV-1 Tg and F344 control rats Potentiation of both the amplitude and dose response (T80) of the signal in control slices exposed to cocaine was observed, which gradually returned to baseline after cocaine washout. However, neither amplitude nor dose response (T80) of the signal was affected by cocaine exposure in the striatum of HIV-1 Tg rats.

**Fig 2:**
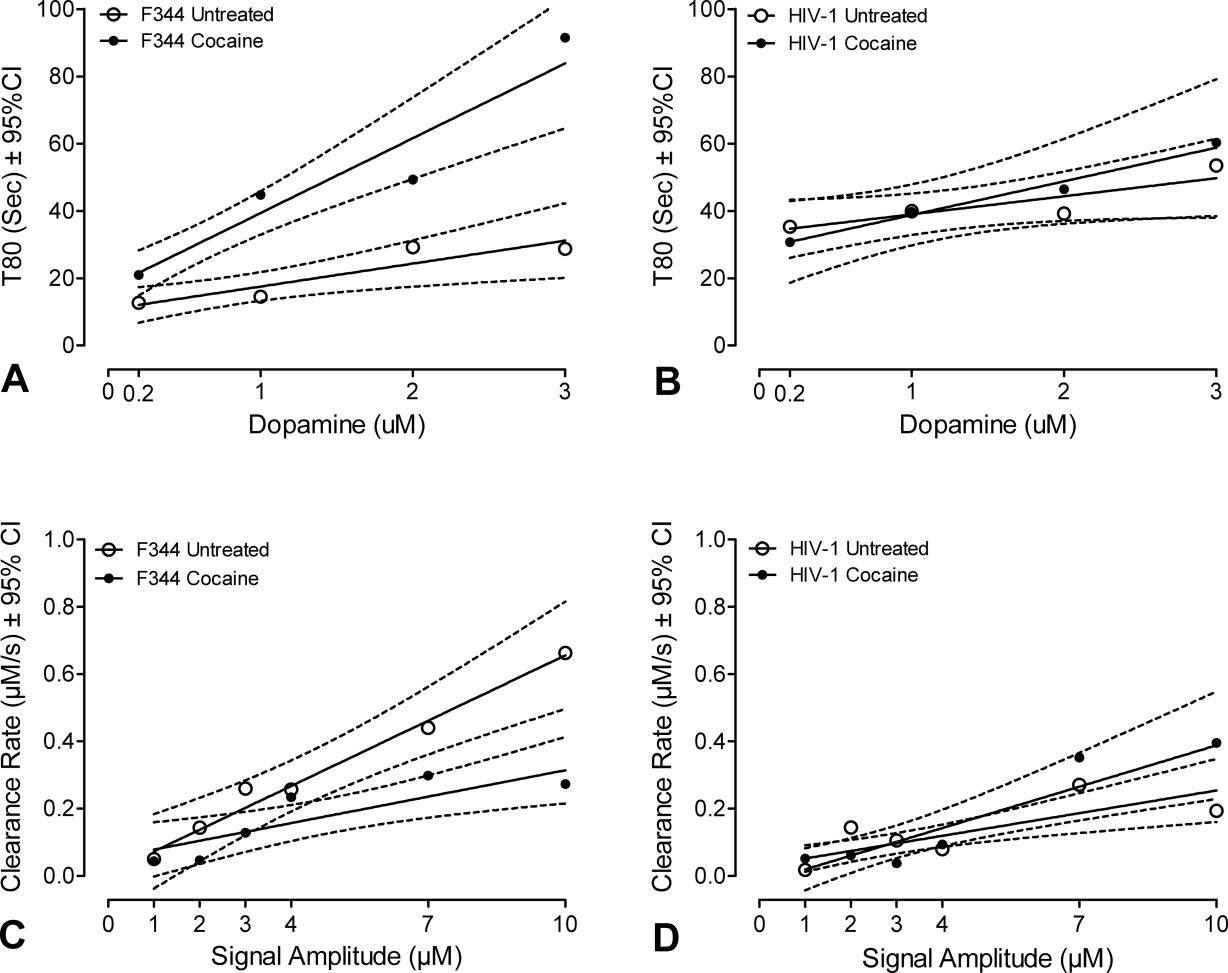
Clearance rate of dopamine was impaired in the HIV-1 Tg rat striatum. Mean T80 values are presented as a function dopamine concentration. (**A**) Dose-response of dopamine in the presence of cocaine results in an increased T80 in control rats (p≤0.04), (**B**) but not HIV-1 transgenic rats; (p≤0.44). Mean clearance rates are presented as a function of signal amplitude. (**C)** Cocaine significantly alters dopaminergic clearance rate in F344/N control rats (p≤0.03). **(D)** Addition of cocaine does not significantly alter the clearance rate of dopamine in HIV-1 transgenic rats (p = 0.13). Data are represented for 10 HIV-1 and 8 control animals; all recordings from each animal were averaged together for the analysis of that animal.

Fig 2 summarizes the effects of cocaine exposure on the signal parameters in the striatum of control and HIV-1 Tg rats, respectively. The time course of the cocaine-evoked signal was markedly different in the HIV-1 striatum relative to controls (Fig 2A and 2B). Although the time course was significantly increased in striatal slices from control rats exposed to cocaine (β_1_ = 6.8±4.7 vs. 22.2±7.8, X±95%CI; F_1,100_ = 4.3, p≤0.04; Fig 2A), it was not significantly altered by cocaine in HIV-1 Tg slices (β_1_ = 5.8±5.6 vs. 10.2±10.2, X±95%CI; F _1, 76_ < 1.0, p = 0.44; Fig 2B), suggesting that the HIV-1 Tg slices were non-responsive to cocaine exposure over the entire amplitude range.

DA clearance in control animals occurred as a linear function of signal amplitude in the striatum of animals exposed, or not exposed, to cocaine (r^2^ = 0.98 and 0.81 for the control and cocaine, respectively), with the slope of the line significantly lower in control slices exposed to cocaine (β_1_ = 0.065±0.025 vs. 0.026±0.016, X±95%CI, for the untreated control slices and cocaine-exposed slices, respectively; F_1,60_ = 5.2, p≤0.027; Fig 2C). As demonstrated previously, our results show that cocaine significantly diminished the clearance rate in control slices; however, HIV-1 Tg slices had an altered response to the addition of cocaine. DA clearance linearly increased as a function of signal amplitude for HIV-1 Tg slices independent of cocaine exposure. While the clearance rate also noticeably potentiates in HIV-1 Tg slices exposed to cocaine, this finding is not statistically significant. (β_1_ = 0.022±0.013 vs. 0.041±0.021, X±95%CI, for the untreated control slices and cocaine-exposed slices, respectively, F _1, 79_ = 3.1, p≤0.12; Fig 2D).

### Dendritic spines of medium spiny neurons

**Fig 3:**
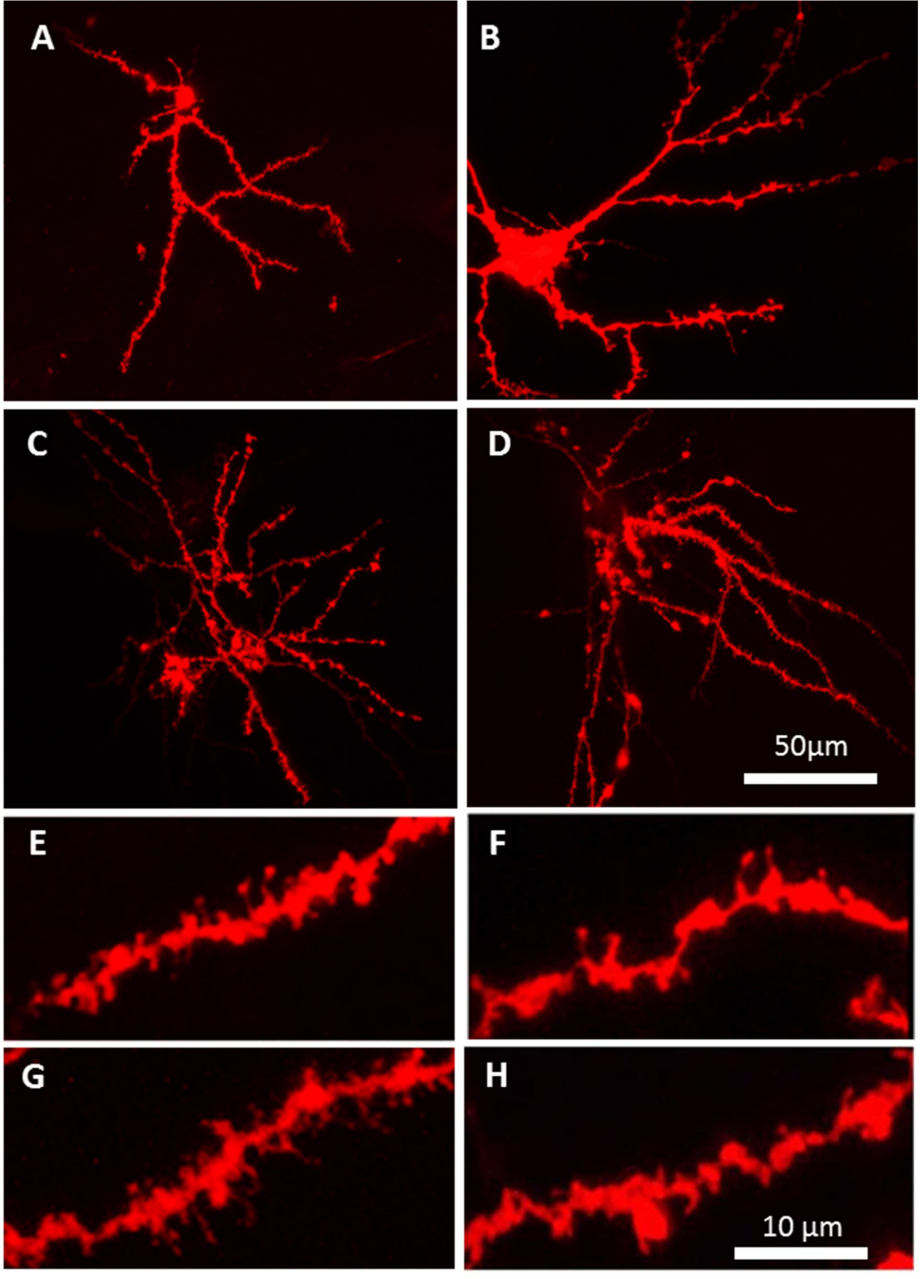
Dendritic spines on medium spiny neurons in the nucleus accumbens. Images illustrating the morphological differences between groups. **(A)** Control MSN exhibited a high number of dendritic spines following diolistic labeling. **(B)** Control MSNs exposed to cocaine exhibited a marked a shift to longer spines. **(C)** HIV-1 Tg untreated slices and **(D)** and HIV-1 Tg slices exposed to cocaine contain medium spiny neurons with shorter spines. **(E-H)** Magnification of dendrite segments from neurons in a-d. HIV-1 Tg dendrites had reductions in dendritic diameter and alterations in spine morphology, with shorter, stubbier, spines. All photomicrographs are representative maximum intensity projections of confocal Z-stacks of medium spiny neurons in the nucleus accumbens. Both representative images and zoom images were taken at 60 X with a Nikon TE-2000 confocal microscope containing ~150–350 Z-sectioned planes, Z-step interval of 150 nm.

Following diolistic labeling, medium spiny neurons in control slices exhibit robust dendritic spine staining extending throughout the dendritic network (Fig 3A and 3E), with cocaine-exposed control neurons exhibiting reductions in spine length (Fig 3B and 3F). In contrast, dendritic spine staining in HIV-1 Tg neurons was reduced, with notable reductions in tertiary dendritic branches (Fig 3C and 3D). HIV-1 Tg neurons (Fig 3G–3H) had reductions in mushroom-type spines (large spine head and high head diameter/neck diameter ratio) (Fig 3G), especially after cocaine exposure (Fig 3H). Cocaine exposure resulted in spine compartmentalization in both control (Fig 3F) and HIV-1 Tg (Fig 3H) neurons, with clear reductions in dendritic diameter between synaptic boutons.

**Fig 4:**
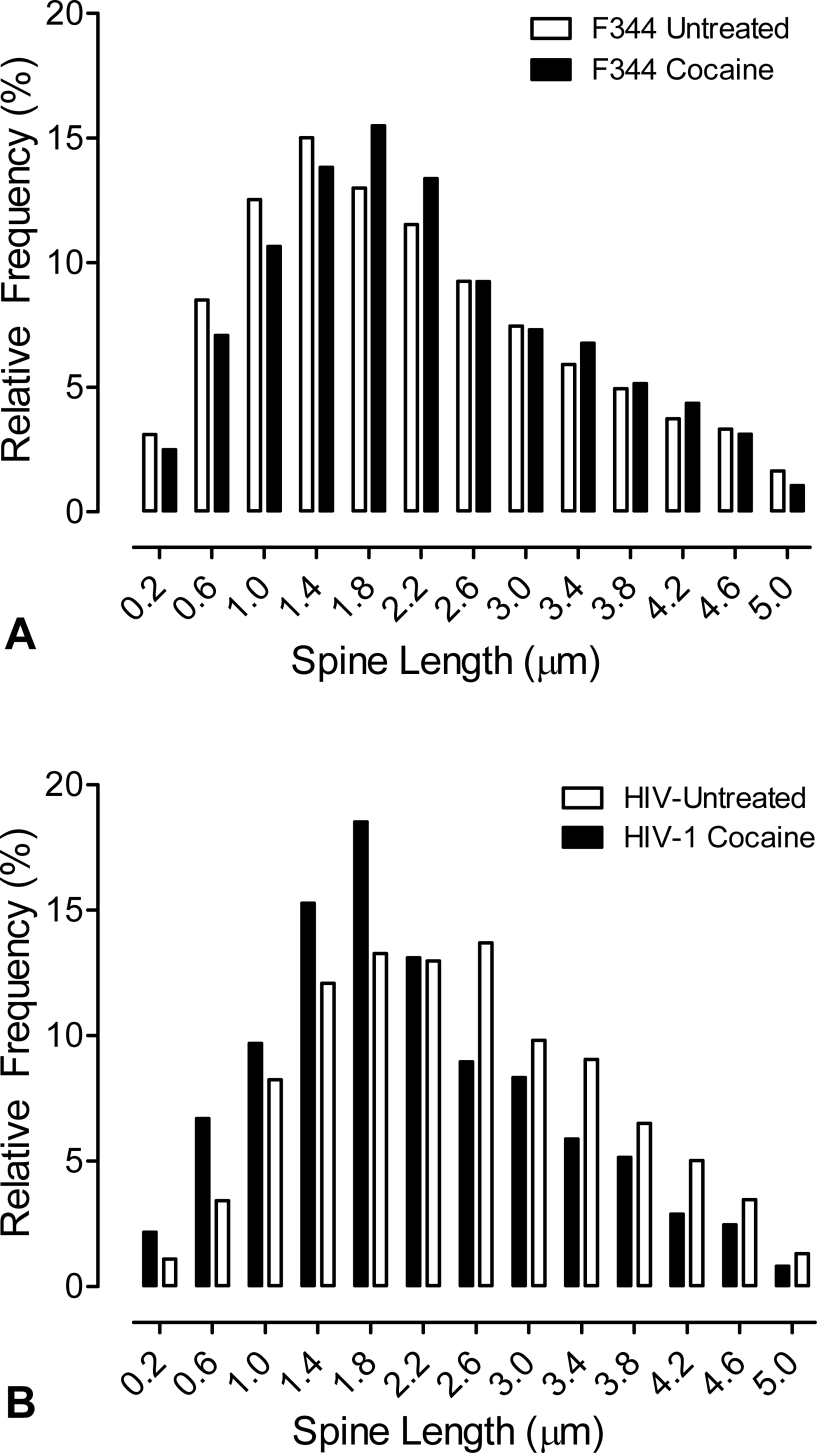
Cocaine differentially alters dendritic spine length. An acute exposure of striatal drug slices to drug challenge was used to determine the neuroplastic response to cocaine. **(A-B)** Relative frequency distributions of dendritic spine length of the MSNs of the nucleus accumbens as a function of cocaine treatment. Length of dendritic spines of the MSNs in the nucleus accumbens increase in the control condition when exposed to cocaine (H(3) = 8.9, N = 17, p_adj_≤0.05); cocaine exposure of HIV-1 Tg MSNs were significantly shifted to shorter spines as a function of cocaine treatment. n = 4–5 animals/group, 1–2 neurons/animal were counted per animal yielding a total of 4,941 spines for analysis of the effect of cocaine in the slices from the F344/N group and a total of 4,708 spines or analysis of the effect of cocaine in the slices from the HIV-1 transgenic group.

Analysis of dendritic spine length showed a differential effect of cocaine on control and HIV-1 MSNs. (Fig 4). Exposure of control (F344) slices to cocaine resulted in a population shift to longer spines (X^2^ = 0.015), whereas exposure of HIV-1 slices to cocaine led to an increased frequency of shorter spines (X^2^ = 0.001).

## Discussion

DA reuptake was decreased in striatal slices from HIV-1 Tg rats, relative to controls. This decrease in uptake was profound, in that the addition of cocaine to slices was not capable of further decreasing the DA reuptake in HIV-1 Tg rats, suggesting profound DAT dysfunction in these animals. Additionally, differential responses to cocaine were found in MSN spine length. In control slices, cocaine treatment increased the population of long spines. In contrast, cocaine treatment increased the frequency of short spines in HIV-1 Tg MSNs. Collectively, these findings indicate profound dysfunction in the striatum of HIV-1 Tg animals and differential response to cocaine challenge between control and HIV-1 Tg MSNs.

The dopaminergic system is significantly altered by exposure to HIV-1 Tat protein [5–7,28–30]. Tat protein can bind directly to the DAT [7,9], inhibiting the reuptake of dopamine, and promoting DAT internalization. Tat-induced conformational changes in DAT increase the binding affinity of cocaine to the transporter protein [6]. Recent studies have demonstrated that interactions of cocaine with DAT may contribute to the cocaine-mediated increase in HIV-1 Tat protein neurotoxicity. Specifically, inhibition of DA uptake and DAT-specific ligand binding was reported in cell cultures exposed to recombinant HIV-1 Tat [10,32] and subtoxic doses of cocaine were found to augment the neurotoxic effects of Tat in primary cultures of rat fetal midbrain neurons [8,32,33]. Combined with cocaine, the converse is also true, as even subtoxic concentrations of Tat induced neuronal degeneration [32]. Although cocaine enhanced Tat-induced toxicity in midbrain cell cultures, the DAT-selective inhibitor, WIN 35428, which binds to different domains of DAT, did not affect Tat neurotoxicity and represents a different ability to mimic synergistic toxicity of cocaine and HIV-1 Tat [8,32]. The modulation of DAT in response to HIV-1 proteins (other than Tat) should be further explored.

Human brain cell cultures infected with HIV-1 display decreased spine density, length and area compared to non-infected controls [34]. Additionally, human neurons incubated with cultured HIV-1 infected astrocytes also display reduced dendritic spine density [35]. Primary neuronal cell culture exposed to both HIV-1 Tat and cocaine resulted in a significant reduction in synaptic integrity, relative to either Tat or cocaine alone[22]. Consistent with previous studies, cocaine potentiated the damaging effects of Tat on synaptic structures [22]. Moreover, the synaptic damaging dose of HIV-1 Tat was a four-fold lower concentration than that previously shown to cause neuronal death [33]. Bertrand et al. (2015) found that primary neuronal cell culture exposed to both HIV-1 Tat and cocaine resulted in a significant reduction in synaptic integrity relative to either HIV-1 Tat or cocaine alone [22].

Tat [9,36] and cocaine both inhibit DAT function and increase oxidative stress [37,38]. Subtoxic doses of cocaine enhance Tat-mediated production of oxidative stress biomarkers such as ROS, intracellular free radical production and protein oxidation, exacerbation of mitochondrial depolarization, neurotoxicity, and cell death [37,38]. The protective action of the D1 receptor selective antagonist, SCH 23390, against Tat and Tat+cocaine *in vitro* suggested that concurrent changes in DA homeostasis induced by Tat and cocaine may contribute to their combined toxicity [37,38,39]. Survival of Tat-exposed neurons was significantly improved when exposed to antioxidants [37], suggesting therapeutic potential for antioxidants in protecting DAT function.

MSN dendritic spine length increased following cocaine exposure, but only in the control condition, as the MSN spines from HIV-1 Tg nucleus accumbens decreased in response to acute cocaine exposure. Such differential responses to cocaine challenge may reflect a diminished capacity for neuroplastic responses of medium spiny neurons in the striatum of HIV-1 Tg animals. This lack of plasticity in HIV-1 Tg slices is consistent with our prior findings of a population shift to shorter, stubbier medium spiny neurons in the nucleus accumbens of HIV-1 Tg female rats [3]. Stubby dendritic spines have little or no spine neck and display reduced synaptic contact area [40,41,42]. Within the MSN population of the nucleus accumbens, approximately 70% of dopaminergic synapses occur on the dendritic spine neck [43]. Thus, reduction in MSN dopaminergic innervation (i.e., an increase in the prevalence of short, stubby spines) may alter the response to cocaine in HIV-1 Tg animals. Coupled with our previous work highlighting Tat-induced allosteric modulation of DAT [7,9], these current results support cocaine-mediated synaptoplasticity inhibition in HIV-1 Tg animals via a dopaminergic mechanism.

Elevated dopaminergic tone in mesolimbic dopaminergic circuits that project through the ventromedial striatum is linked strongly to the reinforcing effects of drugs and drug addiction [44,45]. Recent studies indicate that a similar outcome occurs following exposure to Tat, which can potentiate behaviors mediated by cocaine reward [46]. These results demonstrate that both central expression of HIV Tat-potentiated motivation for cocaine and Tat modulation of dopaminergic activity within the CNS contribute to its capacity to potentiate cocaine’s psychomotor effects [46]. As such, indirect changes to mesolimbic dopaminergic tone via neurotoxic effects of Tat in DA-rich brain regions may exacerbate cocaine-induced psychostimulation [47]. However, direct actions of Tat are more likely to underlie these effects, as opposed to indirect effects of neurotoxic brain disorganization. These data, in parallel with previous findings of Tat-mediated dopaminergic activity and/or sensitization to cocaine’s effects [5,6], suggest that exposure to Tat protein may influence the psychostimulant and rewarding effects of cocaine and increase the likelihood of relapse in abstinent subjects with a history of cocaine use [46].

The current study provides evidence for dysfunction of the dopamine transporter in mediating dopamine reuptake in female HIV-1 Tg rats and in producing altered responses to cocaine. Collectively, dysfunction of the DAT reuptake and altered responses of the nucleus accumbens MSN spine populations suggest a functional disruption of the cocaine reward circuitry within the female HIV-1 Tg rat. Alterations in critical reward pathways in female HIV-1 Tg animals may provide evidence of an interaction of cocaine and HAND in females, although further studies, both in intact female animals and in humans, are necessary to fully corroborate this claim. The interactive effects of HIV-1 and cocaine on synaptic integrity *in vitro* [22] suggest that HIV-1 Tat protein and/or cocaine may alter dendritic spines. Shifts in dendritic spines, as a result of cocaine and/or HIV-1 interactions, may be one key change in the striatum/nucleus accumbens region as a result of excessive dopamine exposure following DAT dysfunction.

## Abbreviations

HIV: human immunodeficiency virus
COC: Cocaine HCl
Tg: Transgenic
DA: Dopamine
DAT: Dopamine transporter
MSNs: Medium spiny neurons
HAND: HIV associated neurocognitive disorders
FSCV: Fast scan cyclic voltammetry
EST: Eastern Standard Time
aCSF: Artificial cerebrospinal fluid T80, Seconds between peak maximum and time where level has decreased 80% from maximum
Tc: Clearance rate (Tc, in mM/sec) defined by the change in DA amplitude between the T20 and T60 time points (e.g., the slope of the linear portion of the decay curve)

## References

[1] Maki PM, Martin-Thormeyer E. HIV, cognition and women. Neuropsychol Rev. 2009; 19: 204–214. doi: 10.1007/s11065-009-9093-2 19430907 10.1007/s11065-009-9093-2 PMC3716452

[2] Meyer VJ, Rubin LH, Martin E, Weber KM, Cohen MH, Golub ET, et al HIV and recent illicit drug use interact to affect verbal memory in women. J Acquir Immune Defic Syndr. 2013; 63: 67–76. doi: 10.1097/QAI.0b013e318289565c 23392462 10.1097/QAI.0b013e318289565c PMC3628722

[3] Roscoe RF Jr., Mactutus CF, Booze RM. HIV-1 transgenic female rat: synaptodendritic alterations of medium spiny neurons in the nucleus accumbens. J Neuroimmune Pharmacol. 2014; 9: 642–653. doi: 10.1007/s11481-014-9555-z 25037595 10.1007/s11481-014-9555-z PMC4440570

[4] Royal W III, Cherner M, Burdo TH, Umlauf A, Letendre SL, Jumare J, et al (2016) Associations between Cognition, Gender and Monocyte Activation among HIV Infected Individuals in Nigeria. PLoS ONE 11(2): e0147182 https://doi.org/10.1371/journal.pone.0147182 Bwala S, Okwuasaba K, Eyzaguirre LM, Akolo C 26829391 10.1371/journal.pone.0147182 PMC4734765

[5] Ferris MJ, Frederick-Duus D, Fadel J, Mactutus CF, Booze RM. Hyperdopaminergic tone in HIV-1 protein treated rats and cocaine sensitization. J Neurochem. 2010; 115: 885–896. JNC6968 [pii]; doi: 10.1111/j.1471-4159.2010.06968.x 20796175 10.1111/j.1471-4159.2010.06968.x PMC4041991

[6] Harrod SB, Mactutus CF, Fitting S, Hasselrot U, Booze RM. Intra-accumbal Tat1-72 alters acute and sensitized responses to cocaine. Pharmacol Biochem Behav. 2008; 90: 723–729. S0091-3057(08)00210-4 [pii]; doi: 10.1016/j.pbb.2008.05.020 18582493 10.1016/j.pbb.2008.05.020 PMC2703478

[7] Zhu J, Ananthan S, Mactutus CF, Booze RM. Recombinant human immunodeficiency virus-1 transactivator of transcription1-86 allosterically modulates dopamine transporter activity. Synapse 2011; 65: 1251–1254. doi: 10.1002/syn.20949 21538554 10.1002/syn.20949 PMC3676522

[8] Aksenov MY, Aksenova MV, Silvers JM, Mactutus CF, Booze RM. Different effects of selective dopamine uptake inhibitors, GBR 12909 and WIN 35428, on HIV-1 Tat toxicity in rat fetal midbrain neurons. Neurotoxicology 2008; 29: 971–977. S0161-813X(08)00107-1 [pii]; doi: 10.1016/j.neuro.2008.06.003 18606182 10.1016/j.neuro.2008.06.003 PMC4205582

[9] Midde NM, Huang X, Gomez AM, Booze RM, Zhan CG, Zhu J. Mutation of tyrosine 470 of human dopamine transporter is critical for HIV-1 Tat-induced inhibition of dopamine transport and transporter conformational transitions. J Neuroimmune Pharmacol. 2013; 8: 975–987. doi: 10.1007/s11481-013-9464-6 23645138 10.1007/s11481-013-9464-6 PMC3740080

[10] Wallace DR, Dodson S, Nath A, Booze RM. Estrogen attenuates gp120- and tat1-72-induced oxidative stress and prevents loss of dopamine transporter function. Synapse 2006; 59: 51–60. doi: 10.1002/syn.20214 16237680 10.1002/syn.20214

[11] Hu S, Sheng WS, Lokensgard JR, Peterson PK, Rock RB. Preferential sensitivity of human dopaminergic neurons to gp120-induced oxidative damage. J Neurovirol. 2009; 15: 401–410. doi: 10.3109/13550280903296346 20175694 10.3109/13550280903296346 PMC7580554

[12] Gelman BB, Spencer JA, Holzer CE III, Soukup VM. Abnormal striatal dopaminergic synapses in national NeuroAIDS tissue consortium subjects with HIV encephalitis. J Neuroimmune Pharmacol. 2006;1: 410–420 doi: 10.1007/s11481-006-9030-6 18040813 10.1007/s11481-006-9030-6

[13] Vera JH, Guo Q, Cole JH, Boasso A, Greathead L, Kelleher P, Rabiner EA, Kalk N, Bishop C, Gunn RN, Matthews PM, Winston A. Neuroinflammation in treated HIV-positive individuals: A TSPO PET study. Neurology 2016; 86: 1425–1432. WNL.0000000000002485 [pii]; doi: 10.1212/WNL.0000000000002485 26911637 10.1212/WNL.0000000000002485 PMC4831035

[14] Royal W III, Zhang L, Guo M, Jones O, Davis H, Bryant JL. Immune activation, viral gene product expression and neurotoxicity in the HIV-1 transgenic rat. J. Neuroimmunology 2012;247: 16–24. doi: 10.1016/j.jneuroim.2012.03.015 22503372 10.1016/j.jneuroim.2012.03.015 PMC3351529

[15] Homji NF, Mao X, Langsdorf EF, Chang SL. Endotoxin-induced cytokine and chemokine expression in the HIV-1 transgenic rat. J. Neuroinflammation 2012;9 doi: 10.1186/1742-2094-9-3 10.1186/1742-2094-9-3 PMC3322344 22216977

[16] Lee DE, Yue X, Ibrahim WG, Lentz MR, Peterson KL, Jagoda EM, et al Lack of neuroinflammation in the HIV-transgenic rat: an [18F]-DPA714 PET imaging study. J. Neuroinflammation 2015; 12: Open access. doi: 10.1186/s12974-015-0390-9 10.1186/s12974-015-0390-9 PMC4574011 26377670

[17] Moran LM, Fitting S, Booze RM, Webb KM, Mactutus CF. Neonatal intrahippocampal HIV-1 protein Tat1-86 injection: neurobehavioral alterations in the absence of increased inflammatory cytokine activation. International Journal of Developmental Neuroscience 2014;28:195–203. doi: 10.1016/j.ijdevneu.2014.09.004 10.1016/j.ijdevneu.2014.09.004 PMC4268159 25285887

[18] Zou C, Shi Y, Ohli J, Schuller U, Dorostkar MM, Herms J. Neuroinflammation impairs adaptive structural plasticity of dendritic spines in a preclinical model of Alzheimer's disease. Acta Neuropathol. 2016; 131: 235–246. 10.1007/s00401-015-1527-8 [pii]. doi: 10.1007/s00401-015-1527-8 26724934 10.1007/s00401-015-1527-8 PMC4713725

[19] Winston CN, Noel A, Neustadtl A, Parsadanian M, Barton DJ, Chellappa D, et al Dendritic Spine Loss and Chronic White Matter Inflammation in a Mouse Model of Highly Repetitive Head Trauma. Am J Pathol. 2016; 186: 552–567. S0002-9440(15)00663-X [pii]; doi: 10.1016/j.ajpath.2015.11.006 26857506 10.1016/j.ajpath.2015.11.006 PMC4816714

[20] Le Y, Liu S, Peng M, Tan C, Liao Q, Duan K, et al Aging differentially affects the loss of neuronal dendritic spine, neuroinflammation and memory impairment at rats after surgery. PLoS One 2014; 9: e106837 PONE-D-14-25996 [pii]. doi: 10.1371/journal.pone.0106837 25198176 10.1371/journal.pone.0106837 PMC4157839

[21] Jurgens HA, Amancherla K, Johnson RW. Influenza infection induces neuroinflammation, alters hippocampal neuron morphology, and impairs cognition in adult mice. J Neurosci. 2012; 32: 3958–3968. 32/12/3958 [pii]; doi: 10.1523/JNEUROSCI.6389-11.2012 22442063 10.1523/JNEUROSCI.6389-11.2012 PMC3353809

[22] Bertrand SJ, Hu C, Aksenova MV, Mactutus CF, Booze RM. HIV-1 Tat and cocaine mediated synaptopathy in cortical and midbrain neurons is prevented by the isoflavone Equol. Front Microbiol. 2015; 6: 894 doi: 10.3389/fmicb.2015.00894 26441850 10.3389/fmicb.2015.00894 PMC4561964

[23] Booze RM, Wood ML, Welch MA, Berry S, Mactutus CF. Estrous cyclicity and behavioral sensitization in female rats following repeated intravenous cocaine administration. Pharmacol Biochem Behav. 1999; 64: 605–610. S0091-3057(99)00154-9 [pii]. 10548278 10.1016/s0091-3057(99)00154-9

[24] Gerhardt GA, Hoffman AF. Effects of recording media composition on the responses of Nafion-coated carbon fiber microelectrodes measured using high-speed chronoamperometry. J Neurosci Methods 2001; 109: 13–21. S0165-0270(01)00396-X [pii]. 11489295 10.1016/s0165-0270(01)00396-x

[25] Hoffman AF, Gerhardt GA. Differences in pharmacological properties of dopamine release between the substantia nigra and striatum: an in vivo electrochemical study. J Pharmacol Exp Ther. 1999; 289: 455–463. 10087038

[26] Burmeister J.J., Gerhardt GA. In Vivo voltammetry for chemical analysis of the nervous system. Encyclopedia of Analytical Chemistry Meyers RA Ed. 2016; 710–731.

[27] John CE, Jones SR. Voltammetric characterization of the effect of monoamine uptake inhibitors and releasers on dopamine and serotonin uptake in mouse caudate-putamen and substantia nigra slices. Neuropharmacology 52: 1596–1605. S0028-3908(07)00063-9 [pii]; doi: 10.1016/j.neuropharm.2007.03.004 17459426 10.1016/j.neuropharm.2007.03.004 PMC2041899

[28] Jones SR, Lee TH, Wightman RM, Ellinwood EH (1996) Effects of intermittent and continuous cocaine administration on dopamine release and uptake regulation in the striatum: in vitro voltammetric assessment. Psychopharmacology 2007; (Berl) 126: 331–338. 10.1007/BF02247384 8878349

[29] Seabold GK, Daunais JB, Rau A, Grant KA, Alvarez VA. DiOLISTIC labeling of neurons from rodent and non-human primate brain slices. J Vis Exp. 2010; 2081 [pii]; doi: 10.3791/2081 10.3791/2081 PMC3156079 20644510

[30] Yuste R. Dendritic spines and distributed circuits. Neuron 2011; 71: 772–781. S0896-6273(11)00651-9 [pii]; doi: 10.1016/j.neuron.2011.07.024 21903072 10.1016/j.neuron.2011.07.024 PMC4071954

[31] Bae J, Sung BH, Cho IH, Kim SM, Song WK. NESH regulates dendritic spine morphology and synapse formation. PLoS One 2012; 7: e34677 PONE-D-12-01126 [pii]. doi: 10.1371/journal.pone.0034677 22485184 10.1371/journal.pone.0034677 PMC3317636

[32] Aksenova MV, Silvers JM, Aksenov MY, Nath A, Ray PD, Mactutus CF, et al HIV-1 Tat neurotoxicity in primary cultures of rat midbrain fetal neurons: changes in dopamine transporter binding and immunoreactivity. Neurosci Lett. 2006; 395: 235–239. S0304-3940(05)01273-5 [pii]; doi: 10.1016/j.neulet.2005.10.095 16356633 10.1016/j.neulet.2005.10.095

[33] Kendall SL, Anderson CF, Nath A, Turchan-Cholewo J, Land CL, Mactutus CF, et al Gonadal steroids differentially modulate neurotoxicity of HIV and cocaine: testosterone and ICI 182,780 sensitive mechanism. BMC Neurosci. 2005; 6: 40 1471-2202-6-40 [pii]; doi: 10.1186/1471-2202-6-40 15943860 10.1186/1471-2202-6-40 PMC1177959

[34] Kurapati KR, Atluri VS, Samikkannu T, Nair MP. Ashwagandha (Withania somnifera) reverses beta-amyloid1-42 induced toxicity in human neuronal cells: implications in HIV-associated neurocognitive disorders (HAND). PLoS One 2013; 8: e77624 PONE-D-13-22227 [pii]. doi: 10.1371/journal.pone.0077624 24147038 10.1371/journal.pone.0077624 PMC3797707

[35] Atluri VS, Kanthikeel SP, Reddy PV, Yndart A, Nair MP. Human synaptic plasticity gene expression profile and dendritic spine density changes in HIV-infected human CNS cells: role in HIV-associated neurocognitive disorders (HAND). PLoS One 2013; 8: e61399 PONE-D-13-01650 [pii]. doi: 10.1371/journal.pone.0061399 23620748 10.1371/journal.pone.0061399 PMC3631205

[36] Ferris MJ, Frederick-Duus D, Fadel J, Mactutus CF, Booze RM. The human immunodeficiency virus-1-associated protein, Tat1-86, impairs dopamine transporters and interacts with cocaine to reduce nerve terminal function: a no-net-flux microdialysis study. Neuroscience 2009; 159: 1292–1299. S0306-4522(09)00044-X [pii]; doi: 10.1016/j.neuroscience.2009.01.024 19344635 10.1016/j.neuroscience.2009.01.024 PMC2715946

[37] Aksenov MY, Aksenova MV, Nath A, Ray PD, Mactutus CF, Booze RM. Cocaine-mediated enhancement of Tat toxicity in rat hippocampal cell cultures: the role of oxidative stress and D1 dopamine receptor. Neurotoxicology 2006; 27: 217–228. S0161-813X(05)00181-6 [pii]; doi: 10.1016/j.neuro.2005.10.003 16386305 10.1016/j.neuro.2005.10.003

[38] Fitting S, Booze RM, Mactutus CF. HIV-1 proteins, Tat and gp120, target the developing dopamine system. Curr HIV Res. 2015; 13: 21–42. CHIVR-EPUB-64725 [pii]. 25613135 10.2174/1570162x13666150121110731 PMC4467793

[39] Silvers JM, Aksenova MV, Aksenov MY, Mactutus CF, Booze RM. Neurotoxicity of HIV-1 Tat protein: involvement of D1 dopamine receptor. Neurotoxicology 2007; 28: 1184–1190. S0161-813X(07)00159-3 [pii]; doi: 10.1016/j.neuro.2007.07.005 17764744 10.1016/j.neuro.2007.07.005 PMC2957183

[40] Harris KM, Kater SB. Dendritic spines: cellular specializations imparting both stability and flexibility to synaptic function. Annu Rev Neurosci. 1994; 17: 341–371. doi: 10.1146/annurev.ne.17.030194.002013 8210179 10.1146/annurev.ne.17.030194.002013

[41] Schmidt H, Eilers J. Spine neck geometry determines spino-dendritic cross-talk in the presence of mobile endogenous calcium binding proteins. J Comput Neurosci. 2009; 27: 229–243. doi: 10.1007/s10827-009-0139-5 19229604 10.1007/s10827-009-0139-5

[42] Shepherd GM, Pologruto TA, Svoboda K. Circuit analysis of experience-dependent plasticity in the developing rat barrel cortex. Neuron 2003; 38: 277–289. S0896627303001521 [pii]. 12718861 10.1016/s0896-6273(03)00152-1

[43] Zahm DS. An electron microscopic morphometric comparison of tyrosine hydroxylase immunoreactive innervation in the neostriatum and the nucleus accumbens core and shell. Brain Res. 1992; 575: 341–346. 0006-8993(92)90102-F [pii]. 1349255 10.1016/0006-8993(92)90102-f

[44] Di CG, Bassareo V, Fenu S, De Luca MA, Spina L, Cadoni C, et al Dopamine and drug addiction: the nucleus accumbens shell connection. Neuropharmacology 2004; 47 Suppl 1: 227–241. S0028390804002199 [pii]; doi: 10.1016/j.neuropharm.2004.06.032 15464140 10.1016/j.neuropharm.2004.06.032

[45] Kalivas PW, Volkow ND. The neural basis of addiction: a pathology of motivation and choice. Am J Psychiatry 2005; 162: 1403–1413. 162/8/1403 [pii]; doi: 10.1176/appi.ajp.162.8.1403 16055761 10.1176/appi.ajp.162.8.1403

[46] Paris JJ, Carey AN, Shay CF, Gomes SM, He JJ, McLaughlin JP. Effects of conditional central expression of HIV-1 tat protein to potentiate cocaine-mediated psychostimulation and reward among male mice. Neuropsychopharmacology 2014; 39: 380–388. npp2013201 [pii]; doi: 10.1038/npp.2013.201 23945478 10.1038/npp.2013.201 PMC3870789

[47] McIntosh S, Sexton T, Pattison LP, Childers SR, Hemby SE. Increased Sensitivity to Cocaine Self-Administration in HIV-1 Transgenic Rats is Associated with Changes in Striatal Dopamine Transporter Binding. J Neuroimmune Pharmacol. 2015; 10: 493–505. doi: 10.1007/s11481-015-9594-0 25749646 10.1007/s11481-015-9594-0 PMC4701048

